# Sexual Intercourse during Pregnancy among Postpartum Women Admitted to the Department of Obstetrics in a Tertiary Care Centre

**DOI:** 10.31729/jnma.8233

**Published:** 2023-10-31

**Authors:** Bibechan Thapa, Smrity Maskey Pradhan, Meenu Maharjan, Aakriti Pandey, Kamana Sen, Shreyasi Karki, Prajwal Pudasaini, Anu Tiwari

**Affiliations:** 1 Department of Paediatrics, KIST Medical College and Teaching Hospital, Mahalaxmi, Lalitpur, Nepal; 2 Department of Gynaecology and Obstetrics, KIST Medical College and Teaching Hospital, Mahalaxmi, Lalitpur, Nepal; 3 Department of Emergency Medicine, KIST Medical College and Teaching Hospital, Mahalaxmi, Lalitpur, Nepal; 4 Department of Dermatology and Venerology, Civil Service Hospital, Minbhawan, Kathmandu, Nepal, Kathmandu, Nepal

**Keywords:** *pregnancy*, *sexual behaviour*, *sexual intercourse*, *sexuality*

## Abstract

**Introduction:**

Pregnancy is a time when women's bodies and minds go through a lot of changes. Sexuality is an important part of a woman's health and well-being, and it often changes during pregnancy. Most women admit that their libido changes in some way during pregnancy. However, the sexuality of a pregnant woman is very individual and influenced by a variety of different factors. This is a very important topic that is often taboo, especially in a male-dominated society, and it needs to be explored more. The aim of this study was to find out the prevalence of sexual intercourse among postpartum women admitted to the Department of Obstetrics in a tertiary care centre.

**Methods:**

A descriptive cross-sectional study was conducted among postpartum women admitted to a tertiary care centre after taking ethical approval from the Institutional Review Committee. The study was carried out from 1 January 2021 to 30 December 2021. Convenience sampling method was used. The point estimate was calculated at a 95% Confidence Interval.

**Results:**

Among 97 pregnant women admitted to the Department of Obstetrics, the prevalence of sexual intercourse was 36 (37.11%) (27.50-46.72, 95% Confidence Interval). A total of 34 (94.44%) were sexually active in the first trimester while 13 (36.11%) and 4 (11.11%) were sexually active in the second trimester and third trimester respectively.

**Conclusions:**

The prevalence of sexual intercourse during pregnancy was lower than other studies done in similar settings.

## INTRODUCTION

Pregnancy is a time when women's bodies and minds go through a lot of changes that affect their lifestyles and practices drastically.^1^ Sexuality is an important part of a woman's health and well-being, and it often changes during pregnancy. Cultural, social, religious, and emotional factors play important roles in it.^1,2^ Most women admit that their libido changes in some way during pregnancy.^3^ However, sexuality is very individual and influenced by different biological, psychological, and sociological factors.^4^ Studies have shown that during pregnancy, there is often a change in a woman's sexuality.^2,5^

This topic is often trivialized as it is thought a taboo in a male-dominated society like Nepal, hence it needs to be explored.^5^

The aim of this study was to find out the prevalence of sexual intercourse among postpartum women admitted to the Department of Obstetrics in a tertiary care centre.

## METHODS

This descriptive cross-sectional study was conducted in the inpatient ward of the Department of Obstetrics at KIST Medical College and Teaching Hospital, Mahalaxmi, Lalitpur, Nepal from 1 January 2021 to 30 December 2021. Ethical approval was taken from the Institutional Review Committee (Reference number: 077/078/11). Postpartum women were included in the study. Women who have recently delivered and who were staying with a sexual partner during pregnancy were included in the study. Convenience sampling method was used. The sample size was calculated using the following formula:


$$\begin{array}{ll} \text{n} & ={\text{Z}}^{2}\times \frac{\text{p}\times \text{q}}{{\text{e}}^{\text{2}}}\\ & =1.96^{2}\times \frac{0.50\times 0.50}{0.10^{2}}\\ & =97 \end{array}$$

Where,

n = minimum required sample size
Z = 1.96 at 95% Confidence Interval (CI)
p = prevalence taken as 50% for maximum sample size calculation
q = 1-p
e = margin of error, 10%

Hence the sample size taken was 97.

A semi-structured questionnaire was made which consisted of two parts: the first part consisted of demographic and obstetric information, and the second part included questions about the sexual practices of women during pregnancy and pre-pregnancy period. The questionnaire was in Nepali language, and only consenting participants were given it to fill out. Women were first briefed about the study and the types of questions included, while still maintaining their privacy. Only those who consented to participate were given the questionnaire, and women were encouraged to answer all of the questions. If a woman refused to answer any questions, she was allowed to do so, and any incomplete questionnaires were discarded. Pretesting was done among 10 women prior to data collection, and any changes made to the questionnaire during this process were incorporated into the final questionnaire.

Data was entered and analysed using IBM SPSS Statistics version 26.0. The point estimate was calculated at a 95% CI.

## RESULTS

Among 97 pregnant women, the prevalence of sexual intercourse was 36 (37.11%) (27.50-46.72, 95% CI).

Among all sexually active women, 34 (94.44%) were sexually active in the first trimester. In the second trimester, 13 (36.11%) women were sexually active while only 4 (11.11%) women were sexually active in the third trimester. Among them, the majority 35 (97.22%), had sexual intercourse more than once a week during pre-pregnant (Table 1).

**Table 1:** Sexual intercourse during pregnancy and pre-pregnancy (n = 36).

Frequency	n (%)
**Sexual intercourse in first trimester**	
More than once a week	10 (27.78)
Less than once a week	24 (66.67)
**Sexual intercourse in second trimester**	
More than once a week	4 (11.11)
Less than once a week	9 (25)
**Sexual intercourse in third trimester**	
More than once a week	-
Less than once a week	4 (11.11)

Regarding initiation of sexual intercourse, 22 (61.11%) responded that it was always the husband who initiate. The majority of women, 20 (55.56%) responded that their desire for sexual intercourse has decreased as compared to the pre-pregnant period. Similarly, 27 (75%) women responded that their satisfaction from sexual intercourse was decreased when compared to the pre-pregnant period (Table 2).

**Table 2:** Women's experiences regarding sexual intercourse during pregnancy (n= 36).

Parameters	n (%)
Initiation of sexual intercourse during pregnancy	
Mostly husband	14 (38.89)
Always husband	22 (61.11)
**Desire for sexual intercourse when compared to pre-pregnant period**	
Same as before	5 (13.88)
Decreased	20 (55.56)
Increased	11 (30.56)
**Satisfaction from sexual intercourse when compared to pre-pregnant period**	
Same as before	4 (11.11)
Decreased	27 (75)
Increased	5 (13.89)

The mean age of pregnant women who were sexually active was 29.33±6.20 years, with a range of 18 to 39 years. The mean duration of marriage was 4.67±3.68 years. Women in the 26-30 age group 10 (27.78%) were sexually active. Additionally, women who have been married for less than 5 years and multiparous women (42.31%) were more sexually active at 21 (58.33%) and 22 (61.11%) (Table 3).

**Table 3:** Socio-demographic characteristics of sexually active women (n = 36).

Socio-demographic characteristics	n (%)
**Age (years)**	
18-20	2 (5.56)
21-25	9 (25)
26-30	10 (27.78)
31-35	7 (19.44)
36-40	8 (22.22)
**Duration of marriage (years)**	
<5	21 (58.33)
≥5	15 (41.67)
**Religion**	
Hindu	24 (66.67)
Buddhist	8 (22.22)
Christian	1 (2.78)
Muslim	3 (8.33)
**Education of women**	
No formal education	-
Primary education	-
Secondary education	6 (16.67)
Higher secondary	13 (36.11)
Bachelors	9 (25)
Master and above	8 (22.22)
**Education of husband**	
No formal education	-
Primary education	-
Secondary education	9 (25)
Higher secondary	5 (13.89)
Bachelors	9 (25)
Master and above	13 (36.11)
**Parity**	
Primipara	14 (38.89)
Multipara	22 (61.11)

Only 6 (16.67%) women have discussed sexual intercourse during pregnancy with their doctors and the majority of women, 19 (52.78%) used the internet and social media as sexual intercourse of information about sexual intercourse during pregnancy (Table 4).

**Table 4:** Sources of information regarding sexual intercourse during pregnancy (n= 36).

Source of information	n (%)
Doctors and health professionals	6 (16.67)
Internet and social media	19 (52.78)
Friends and family	4 (11.11)
Partners	7 (19.44)

## DISCUSSION

According to our study, 36 (37.11%) pregnant women were engaged in sexual activity. In a study from Nigeria, it was found that 171 (98.8%) pregnant women were engaged in sexual activity.^6^ A total of 124 (80%) women were sexually active while pregnant in a study done among Iranian couples.^7^ It was reported 211 (52%) women had sexual intercourse during pregnancy in a study done in Beijing, China.^8^ This significant decrease in sexual intercourse during pregnancy in our study group may be attributed to the cultural practice of the country, lack of awareness that intercourse during pregnancy is not contraindicated unless recommended by their obstetrician, existing strong misbelief that it could cause miscarriage, stillbirth and fetal infections, lack of proper counselling by their health care providers about safe sexual practices during pregnancy, lack of partners being vocal to each other about their sexual expectations and needs during pregnancy due to lack reliable knowledge that causes them to avoid intimate acts during pregnancy.

We studied the age, parity, education level and duration of marriage of the pregnant women with an aim to find out the factors that would be attributable to the variation in sexual practices. The most significant change was found in the educational status of pregnant women. With increasing education levels, there was more involvement in sexual activity as educated women are more likely to search for other sources of information about sexuality, thereby protecting themselves from misconceptions that have been shown to cause sexual dysfunction during pregnancy. Educational level is thought to be one of the predisposing factors associated with personality traits for help-seeking behaviour regarding sexual dysfunction.^6,9^

There is a statistically significant difference in overall sexual frequency between the first, second and third trimesters in our study. The frequency of sexual intercourse in the first trimester was 34 (94.44%), the second trimester was 13 (36.11%) and the third trimester was 4 (11.11%). This could be because of factors such as emotional stress and anxiety regarding the possible complications being increased as the time of delivery approaches. Increased fatigue, shortness of breath, oedema, contractions, fear of harm to the mother and the fetus, decreased libido, a diminished image of one's sexual self, and dyspareunia are other reasons for the decrease in sexual intercourse frequency as the pregnancy progresses.^6,10^ The findings of our study are consistent with a study done in Lisbon, Portugal, where it was found that the first trimester was considered the most frequent period of intercourse (44.7%), followed by the second trimester (35.6%). 55% reported decreased sexual activity in the third trimester of pregnancy.^2^

The findings of the study showed that it was mostly husbands who initiated intercourse during the pregnancy. There is a decrease in the solitary desire as the pregnancy advances in the women. The women also focus more on the unborn baby and set aside the sexual needs of their as well as their partners, especially during the third trimester of the pregnancy.^11^ Apart from this, in Nepal women have limited autonomy over their sexual and reproductive health as husbands exercise most of the control regarding intercourse, pregnancy, childbirth and family planning.^12^ The desire for sexual intercourse compared to the prepregnant state was also found to be low 20 (55.56%). Similar results were found in a study where the mean frequency of intercourse during pregnancy (1.6 times/week) was less than before pregnancy (3.5 times/week) and husbands were the main initiators of sexual activity (44%), only (0.7%) pregnant women initiated sexual intercourse.^5^ The satisfaction from sexual intercourse during pregnancy was also significantly decreased in our study population 27 (75%). This could be attributed to the feeling of guilt regarding sexual relations during pregnancy, altered body image, reduced sense of charm for the spouse, fear of injury to the fetus, fear of abortion, and sexual dysfunction.^13^ While in a Portuguese study, sexual satisfaction remained unchanged at (48.4%) and decreased at (27.7%). Sexual desire remained unchanged in (38.8%) and decreased in (32.5%).^2^

It was found that only 6 (16.67%) consulted about sexual intercourse with their doctors and health professionals. They received most of their sexual information during pregnancy from the internet and social media, friends, family, and partners. This could be because of fear among the health professionals that asking questions of sexual nature would violate the patient's privacy, and questions about sexuality being construed as sexual harassment leading to legal consequences. Similarly, there is also hesitancy from the patient's side to initiate conversations about the highly taboo topic of sex with their healthcare providers.^14^

The major limitation of this study is that it is a singlecentred study and a small sample size was taken so generalisation cannot be done, and as sex and sexual practices are topics that are often taboo, it is likely that many women would be reluctant to provide accurate information.

## CONCLUSIONS

The prevalence of sexual intercourse during pregnancy was lower than other studies done in similar settings. Many pregnant women abstain from sexual activity, and the frequency of sexual intercourse decreases as pregnancy progresses. Higher-educated women are more involved in sexual activity during pregnancy, underscoring the importance of education and awareness about sexuality during pregnancy. Hence, more research is necessary on this topic, and that discussion about it with patients should be encouraged among healthcare providers.
